# Pleural Resident Macrophages and Pleural IRA B Cells Promote Efficient Immunity Against Pneumonia by Inducing Early Pleural Space Inflammation

**DOI:** 10.3389/fimmu.2022.821480

**Published:** 2022-04-14

**Authors:** Alan Bénard, Malgorzata J. Podolska, Franziska Czubayko, Isabella Kutschick, Bettina Klösch, Anne Jacobsen, Elisabeth Naschberger, Maximilian Brunner, Christian Krautz, Denis I. Trufa, Horia Sirbu, Roland Lang, Robert Grützmann, Georg F. Weber

**Affiliations:** ^1^ Department of Surgery, Universitätsklinikum Erlangen, Friedrich-Alexander Universität Erlangen-Nürnberg, Erlangen, Germany; ^2^ Department of Thoracic Surgery, Universitätsklinikum Erlangen, Friedrich-Alexander Universität Erlangen-Nürnberg, Erlangen, Germany; ^3^ Institute of Clinical Microbiology, Universitätsklinikum Erlangen, Friedrich-Alexander Universität Erlangen-Nürnberg, Erlangen, Germany

**Keywords:** pleural resident macrophages, IRA B cells, necroptosis, mincle, pleural space, bacterial airway infection

## Abstract

Airway infection is a major cause of mortality worldwide. The identification of new mechanisms aiding in effective host immune response is therefore required. Here, we show that the specific depletion of the pleural immune cell compartment during bacterial pneumonia resulted in a reduced pulmonary immune response and increased mortality in mice. Bacterial airway infection provoked early pleural space (PS) inflammation characterized by innate response activator (IRA) B cell development and pleural large resident macrophage (LRM) necroptosis, the repopulation of LRMs being driven by cellular proliferation *in situ*. Necroptotic LRMs amplified PS inflammation by stimulating pleural Mincle-expressing macrophages whereas IRA B cells contributed partially to GM-CSF-induced PS inflammation. Upon pulmonary infection, the induction of PS inflammation resulted in reduced bacterial burden whereas the specific depletion of pleural resident macrophages led to increased mortality and bacterial burden and reduced pulmonary immunity. Moreover, mice in which B cells were unable to produce GM-CSF exhibited reduced CD103^+^ dendritic cells and reduced CD4^+^ T cell numbers in the draining lymph node. Altogether, our results describe a previously unrecognized mechanism of pleural space inflammation necessary for effective protection against bacterial airway infection.

## Introduction

Lower respiratory tract infection is the world’s most deadly communicable disease, ranked as the 4th leading cause of death worldwide (WHO statistics 2020) resulting in a significant burden on the global health care systems (1–5). Due to an increase of antibiotic resistance within the last decades, one of the main treatment pillars for bacterial lower respiratory tract infections has been compromised, increasing the risk for exacerbation of the infection and possibly leading to pneumogenic sepsis associated with high morbidity and mortality (6, 7). For instance, hospital-acquired pneumonia, mainly caused by Gram negative bacteria such as *Escherichia coli* (*E.coli*), is the second most common hospital-acquired infection (HAI) in United States and represents 33 to 50% of mortality related to HAI (6). Thus, a better comprehension of the mechanisms protecting the host upon airway infection may lead to novel therapies for the treatment of pneumonia.

The pleural space (PS) is the serosal cavity between the two pulmonary pleurae (parietal and visceral) helping to the optimal functioning of the lungs during breathing. However, pulmonary inflammation may lead to pleural inflammation, which is associated with higher mortality in patients suffering from severe pneumonia (8, 9). PS inflammation is therefore considered to be detrimental (10–12). However, we recently identified pleural innate response activator (IRA) B cells aiding in the protection against pneumonia through the Granulocyte–macrophage colony-stimulating factor (GM-CSF) mediated production of polyreactive emergency IgM (13), suggesting an unexpected contribution of PS immune cells for effective pulmonary immunity against airway infection. In addition to serosal B1 B cells, PS harbors T cells and two subpopulations of resident macrophages (RM): the F4/80^+^ MHCII^lo^ large macrophages (LRMs) derived from embryonic precursor and maintained locally through Gata6 and the less abundant F4/80^lo^ MHCII^+^ small macrophages (SRMs) developed from circulating monocytes (14, 15). The role of pleural resident macrophages is still poorly understood. Previous studies described that pleural resident macrophages were mainly responsible of neutrophil influx into the pleural cavity upon intrapleural administration of carrageenan (16). As well, pleural macrophages are locally programmed to silently clear apoptotic cells (17).

Macrophage-inducible C-type lectin (Mincle) is an innate immune receptor belonging to the C-type lectin receptor family. It is expressed by innate immune cells such as macrophages and neutrophils (18, 19) and sense conserved structural motifs of both microbial and endogenous danger signals resulting in the secretion of immune mediators modulating inflammation and immunity (20). Recent studies reported that Mincle-deficient mice exhibited increased bacterial loads and decreased survival together with strongly dysregulated cytokine responses upon bacterial pneumonia (21, 22) revealing an important role of Mincle during pulmonary immune response. However, little is known about a role of Mincle in the PS.

In this study, we have investigated the role and the composition of the pleural space during *E.coli* airway infection. We show that the specific depletion of the pleural space immune cell compartment during airway infection resulted in reduced protection upon *E. coli* pneumonia. We observed immediate PS inflammation upon pulmonary infection resulting in the rise of pleural IRA B cells and the early increase of pleural LRM necroptosis. In addition, we deciphered that the repopulation of LRM was driven by their local cellular proliferation. We observed that GM-CSF potentiated PS inflammation and that GM-CSF effect was partially associated to IRA B cells. Necroptotic LRMs promoted also PS inflammation in a Mincle-associated manner. We revealed that the induction of PS inflammation by the direct injection of LPS into the PS improved pulmonary immunity and led to reduced bacterial burden upon *E. coli* airway infection whereas the specific depletion of pleural RMs led to a reduced lung immunity. As well, the deletion of GM-CSF in B cells led to reduced migration of CD103^+^ dendritic cells into the tracheobronchial lymph node as well as a reduced number of CD4^+^ T cells in lungs, BAL and their draining lymph node. Collectively, our results reveal that the early immune activation of the PS is important for mounting an effective immune response against bacterial airway infection.

## Material and Methods

### Animals

Wild-type (WT) (Janvier, Le Genest-Saint-Isle, France), *Csf2* ^-/-^ (GM-CSF deficient), µMT (B cell deficient) and *Clec4e* ^-/-^ (Mincle deficient) mice were used in this study. All mice were on C57BL/6 background and were 8-12 weeks old when sacrificed.

### Mixed Bone-Marrow Chimera

Mixed bone-marrow chimera mice were prepared as previously described (13). Briefly, naive WT mice were lethally irradiated (10 Gy). 4-7 h after irradiation, animals were reconstituted with a 1:1 mixture of total bone marrow cells from µMT, WT or *Csf2^-/-^* mice. A total of 4 x 10^6^ cells was injected intravenously. Animals were allowed to recover for a minimum of 8 weeks.

### Injection Models

For intra-nasal injection, mice were under isoflurane anesthesia. Then a maximum of 20 µl of saline solution was gradually released into the nostrils (10 ul in each nostril) with the help of a micropipette. For intra-tracheal injection, a skin incision was performed over 1 cm under isoflurane anesthesia. Then, the trachea was identified and a volume of 50 µl saline was injected progressively with a 0,3ml Syringe (30-gauge needle) directly into the trachea. After the injection we verified that mice had adequate breathing abilities. Skin closure was performed with an Ethilon 5/0 (Johnson and Johnson, New Brunswick, NJ, USA) suture and pain medication (Buprenorphin, 0.1 mg/kg) was injected i.p. (23G Terumo, Leuven, Belgium). For intra-pleural injection using the Inter-Costal Approach of the Pleural Space (ICAPS) model, a right antero-lateral thoracic incision was performed over 2-3 cm under isoflurane anesthesia. Then, the attached muscles from the thoracic wall were dissected. 100µl or 200µl of talc, LPS, GM-CSF, TNFα, Nec-1s, purified pleural B cells or clodronate liposome were injected into the pleural space with a polyethylene catheter (Smith Medicals, Minneapolis, MN, USA) (ID/OD. 0.58x0.96mm) inserted tangentially without damaging the lung parenchyma. After removing the catheter, we controlled the absence of pneumothorax and we verified that mice had adequate breathing abilities. Skin closure was performed with an Ethilon 5/0 suture and pain medication (Buprenorphin, 0.1 mg/kg) was injected i.p.

### Mouse Treatment

Mice were treated i.n., i.t. or i.pls. with 20 µg LPS (Sigma Aldrich, St. Louis, MO, USA), 100 ng recombinant TNFα (Peprotech, Rocky Hill, NJ, USA), 100ng recombinant GM-CSF (Peprotech), 2.5, 5 or 10 × 10^6^ CFU *Escherichia coli* (ATCC) or 7.5x10^6^ PFU HSV-1 in a volume of 15µl saline. For talc experiments, 200 µl of a 5% Talc 0.9% NaCl solution was injected in pleural space 1 week before infection. For the necrostatin (Nec-1s) inhibitor experiment (Biozol, Eching, Germany), 100 µg of Nec-1s was injected in pleural space just before infection. For the zVAD-fmk inhibitor experiment (Invivogen, San Diego, CA, USA), 100 µg of zVAD-fmk was injected in pleural space just before infection. For the pleural RM depletion, 100 µl of clodronate liposome and liposome control (Liposoma B.V.) were injected in pleural space 2 days before infection. For the adoptive transfer of B cells, 10^6^ purified pleural B cells from WT mice in a volume of 100 µl saline was injected in pleural space just before intra-pleural injection of LPS.

### Murine Leukocytes Isolation

After organ harvest, single cell suspensions were obtained as follows: perfused lungs were cut in small pieces and subjected to enzymatic digestion with 450 U/ml collagenase I (Sigma Aldrich), 125 U/ml collagenese IX (Sigma Adrich), 60 U/ml hyaluronidase (Sigma Aldrich), 60 U/ml DNase (Sigma Aldrich) and 20 mM Hepes (Thermo Fisher Scientific, Waltham, MA, USA) for 1 hour (lungs) at 37°C while shaking; the pleural space was flushed with 4 × 1 ml of PBS to retrieve leukocytes; broncho-alveolar lavage (BAL) was performed by flushing the lungs with 2 × 1 ml of PBS to retrieve the infiltrated and resident leukocytes; tracheobronchial lymph node was ground on top of a 40µm filter (Thermo Fisher Scientific) with a syringe plunger. Total viable cell numbers were obtained using Trypan Blue (Carl Roth). Pleural B cells were purified by positive magnetic selection using anti-CD19 microbeads, according to the manufacturer’s instructions (Miltenyi Biotec).

### Bacteria Titration

Bronchoalveolar lavage fluid and non-perfused lung suspension samples were plated on tryptic LB agar (Carl Roth, Karlsruhe, Germany) and incubated at 37°C overnight.

### Pleural Immune Cells Stimulation *Ex Vivo*

Pleural immune cells were cultured in RPMI-1640 GlutaMax supplemented with 10% FCS, 25mM of Hepes, 1 mM sodium pyruvate, 100U/ml of Penicillin-Streptomycin and 20µg/ml of Gentamicin at 37°C in the presence of 5% CO2. Pleural immune cells were stimulated in 12-well plates (106 cells/ml) during 24h by LPS (2µg/ml), Nec-1s (50µM), GM-CSF (20ng/ml) or Trehalose-6,6-dibehenate (TDB, a Mincle agonist) (10µg/ml) (Invivogen). Then supernatants were collected for cytokine measurement. For analyzing the level of phosphorylation of Syk by flow cytometry, PS cells were stimulated during 30min by TDB (10µg/ml).

### Histology

Tissue sections with 4 µm were cut from formalin-fixed paraffin embedded (FFPE) lungs and stained by haematoxylin/eosin. In brief, the sections were dewaxed using two times xylol for 10 min followed by rehydration in a decreasing ethanol row. After a wash in A.d. the sections were stained by haematoxylin Gill-III (1:3 diluted, Merck) for 3.5 min, followed by 10 min warm tap water. Subsequently, staining for 1 min in eosin Y solution (Sigma) and two times washing in A.d. for 5 min followed. Slides were subjected to an increasing ethanol row and finally mounted using VectaMount Permanent Mounting medium (Vector Laboratories).

### Quantitative RT-PCR

Real-time PCR was performed as previously described (23). Briefly, RNA was extracted from cells by RNeasy mini kit (Qiagen, Venlo, Netherlands). Complementary DNA was reverse transcribed from 1 µg total RNA with Moloney murine leukemia virus reverse transcriptase (Thermo Fisher Scientific) using random hexamer oligonucleotides for priming (Thermo Fisher Scientific). The amplification was performed with a Biorad CFX-Connect Real-time-System (Thermo Fisher Scientific) using the PCR SYBR Green sequence detection system (Eurogentec, Seraing, Belgium) or Taqman (Thermo Fisher Scientific). Data were analyzed using the software supplied with the Sequence Detector (Life Technologies). The mRNA encoding for murine *Tnfα*, *Il-1ß*, *Cxcl1*, *Il-6* and *Mincle* were normalized to the hypoxanthine-guanine phosphoribosyltransferase (*Hprt*) mRNA. Gene expression was quantified using the ΔΔCt method. Murine primers used are the followings: *Hprt* forward: 5’-GTTCTTTGCTGACCTGCTGGAT-3’, *Hprt* reverse: 5’-CCCCGTTGACTGATCATTACAG-3’; *Il-1β* forward: 5’-GCCCATCCTCTGTGACTC AT-3’, *Il-1β* reverse: 5’-AGGCCACAGGTATTTTGTCG-3’; *Tnfα* forward: 5’-CAAAATTCGAGTGACAAGCCTGTA-3’, *Tnfα* reverse: 5’-CCA CTTGGTTTGCTACGA-3’; *Cxcl1* forward: 5’- ATCCAGAGCTTGAAGGTGTTG -3’, C*xcl1* reverse: 5’- GTCTGTCTTCTTTCTCCGTTACTT -3’; *Clec4e* forward: 5’- GCTCACCTGGTGGTTATCG-3’, *Clec4e* reverse: 5’- AGGTTTTGTGCGAAAAAGGA-3’., *Il-6* forward: 5’- ATGTTCTCTGGGAAATCGTGGA-3’, *Il-6* reverse: 5’- AGAATTGCCATTGCACAACTCTT-3’.

### Cytokine Detection

Secreted murine TNFα (Biolegend, San Diego, CA, USA), murine IL-6 (Biolegend), murine GM-CSF (Biolegend), human TNF (Biolegend) and human IL-6 (Biolegend) were measured by ELISA according to the manufacturer’s instructions.

### Flow Cytometry

The following antibodies were used for flow cytometric analyses: Mouse: anti-CD19-PE (1D3, BD Biosciences), anti-CD49b-PE (DX5, BD Biosciences), anti-CD90.2-PE (53-2.1, BD Biosciences), anti-B220-PE (RA3-6B2, BD Biosciences), anti-Ter119-PE (Ter-119, BD Biosciences), anti-CD127-PE (SB/199, Biolegend), anti-Gr1-PE (RB6-8C5, Biolegend), anti-CD11b-BUV737 (M1/70, BD Biosciences), anti-CD11b-PE CF594 (M1-70, BD Biosciences), anti-MHCII-PerCP Cy5.5 (AF6-120.1, Biolegend), anti-MHCII-BV711 (M5/114.15.2, BD Biosciences), anti-CD45.2-BV786 (104, BD Biosciences), anti-Ly6C- BV711 (HK1.4, Biolegend), anti-Ly6C-FITC (AL-21, BD Biosciences), anti-CD11c-PerCP Cy5.5 (HL3, Biolegend), anti-F4/80-PE CF594 (BM8, BD Biosciences), anti-F4/80-BV510 (T45-2342, BD Biosciences), anti-Ly6G-BUV395 (1A8, BD Biosciences), anti-CD115- BV510 (T38-320, BD Biosciences), anti-CD115-PE (T38-320, BD Biosciences), anti-B220-BUV737 (RA3-6B3, BD Biosciences), anti-CD3-PE CF594 (17A2, BD Biosciences), anti-CD3-BV650 (145-2C11, BD Biosciences), anti-CD20-BV421 (SA275A11, Biolegend), anti-Mincle-Biotin (1B6, Biozol, Eching, Germany), anti-CD138-PerCP Cy5.5 (281-2, Biolegend), anti-CD103-BV421 (M290/2E7, BD Biosciences), anti-CD93-BUV395 (AA4.1, BD Biosciences), anti-CD43-FITC (S7, BD Biosciences), anti-IgM-BV650 (R6-60:2, BD Biosciences); Human: anti-CD45-BV786 (HI30, BD Biosciences), anti-HLADR-BUV395 (G46-6, BD Biosciences), anti-CD64-PE (10.1, Biolegend), anti-Mincle (OTI3F4, Origene (Rockville, MD, USA)). Zombie Green dye and FITC Annexin V Apoptosis Detection Kit with 7-AAD were used to analyze the death of resident macrophages (Biolegend). Streptavidin-BUV395 (BD Biosciences) was used for Mincle staining. Staining for intracellular phospho-Syk was performed using BD Cytofix/Cytoperm Plus Kit (BD Biosciences). Data were acquired on a Celesta (BD Biosciences) flow cytometer and analyzed with FlowJo 10 (FlowJo LLC, Ashland, OR, USA). The murine cell types were defined as followed: Neutrophils = CD45^+^ CD11b^+^ Ly6G^+^ Ly6C^+^ F4/80^-^; B cells = CD45^+^ B220^+^ MHCII^+^ CD3^-^ F4/80^-^; T cells = CD45^+^ B220^-^ CD3^+^ F4/80^-^ MHCII^-^; Inflammatory macrophages = CD45^+^ CD11b^+^ MHCII^+^ Ly6G^-^ CD115^+^ F4/80^int^ Ly6C^+^; Resident macrophages = CD45^+^ CD11b^+^ MHCII^+^ Ly6G^-^ CD115^+^ F4/80^high^ Ly6C^-^; CD103^+^ DCs = CD45^+^ Lin^-^ (Lin includes CD19, CD49b, CD90, B220, Ter119, CD127) CD11c^+^ MHCII^+^ CD103^+^; IRA B cells = CD45^+^ MHCII^+^ CD19^+^ IgM^+^ CD43^+^ CD138^+^ CD93^+^. The human pleural macrophage was described as followed: CD64^+^ HLADR^+^ CD45^+^.

### Human Pleural Effusion Fluid

The patients who underwent surgery and gave their approval were included in this study. After accessing the pleural space, the pleural fluid was harvested directly using a sterile syringe or a lavage with 100ml 0.9% saline was performed. Fluid has been obtained and processed for flow cytometric analysis of leukocyte surface markers and *ex vivo* stimulation. In addition, after centrifugation the supernatants have been stored at -80°C until further processing.

### Study Approval

Mouse: All animal protocols were approved by the animal review committee from the university hospital Dresden and Erlangen and the local governmental animal committee. Human: The study was performed at the University of Erlangen in Germany. Patients were selected within the framework of the thoracic surgery board. The patients who underwent surgery and provided informed consent were included in this study. The study was performed in agreement with the local ethics review board of the University of Erlangen (UKER 10_16 B; UKER 339_15 Bc; UKER 56_12B; DRKS-ID: DRKS00005376). Patients’ confidentiality was maintained.

### Statistics

Results were expressed as mean ± S.E.M. Statistical tests included paired or unpaired, 2-tailed Student’s t test using Welch’s correction for unequal variances, Dunnett’s multiple comparisons test or Tukey’s multiple comparisons test. P values of 0.05 or less were considered to denote significance. Significant outliers were determined using GraphPad Prism 7.0 software and excluded from the analysis.

## Results

### Pleural Space Cells Protect Against *E. coli* Airway Infection

**Figure 1:**
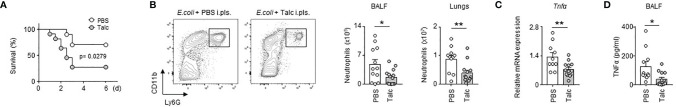
Pleural space contributes to protection upon *E. coli* airway infection. **(A)** Survival curve of mice after *E. coli* i.t. injection in mice that received pleural injection of talc (grey) or PBS (white) 7 days before infection (n=8-9 mice). **(B)** Enumeration of neutrophils in BALF and lungs 4 days after *E. coli* i.t. injection in mice depleted (grey) or not (white) of pleural cells by talc injection (n=11-13 mice). **(C)** Relative mRNA expression of *Tnfα* in lungs 4 days after *E. coli* i.t. injection in mice depleted (grey) or not (white) of pleural cells by talc injection; the expression level was arbitrarily set to 1 for one sample from the PBS group, and the values for the other samples were calculated relatively to this reference (n=11-13 mice). **(D)** TNFα level in BALF 4 days after *E. coli* i.t. injection in mice depleted (grey) or not (white) of pleural cells by talc injection (n=11-13 mice). Data are pooled data from at least 2 independents experiments. Data represent mean ± S.E.M. and were analyzed by Log-rank test or by the two-tailed unpaired t-test. *p<0.05; **p<0.01.

To investigate the role of the PS during airway infection, we specifically and significantly reduced the number of immune cells in the pleural space compartment by intra-pleural injection of talc (**Figure S1**), a technique currently used in the clinic to prevent recurrent malignant pleural effusion (24, 25). Compared to controls, mice treated with talc displayed a higher mortality upon *E. coli* airway infection over time (**Figure 1A**). Moreover, depletion of PS immune cells by talc resulted in a reduction of neutrophil influx in BALF and lungs upon *E. coli i.t.* airway infection (**Figure 1B**) as well as a reduction of the mRNA expression of the pro-inflammatory cytokine *Tnfα* in the lungs (**Figure 1C**). Likewise, a reduction of BALF TNFα levels was observed in mice depleted of PS cells (**Figure 1D**). Collectively, our results suggest an unexpected protective role of the PS immune cell compartment during *E. coli* airway infection.

### Immediate Pleural Space Inflammation Upon *E. coli* Airway Infection

**Figure 2:**
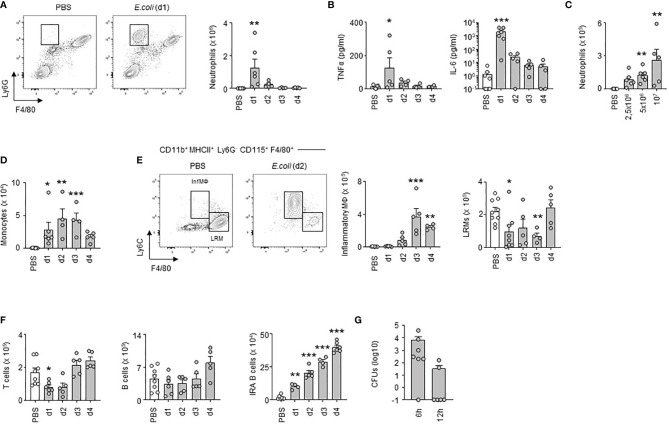
Early inflammation of pleural space upon *E. coli* airway infection. **(A)** Representative dot plots and enumeration of neutrophils in pleural space 1, 2, 3 and 4 days after i.t. infection of 5x10^6^ CFU of *E. coli* or PBS (n=5-7 mice per time point). **(B)** Levels of TNFα and IL-6 in pleural space 1, 2, 3 and 4 days after i.t. infection of 5x10^6^ CFU of *E. coli* or PBS (n=5-7 mice per time point). **(C)** Enumeration of neutrophils in pleural space 4 days after i.t. infection of 0, 2.5, 5 or 10x10^6^ CFU of *E. coli* (n=5-6 mice). **(D–F)** Enumeration of monocytes **(D)**, inflammatory and large resident macrophages **(E)**, T cells, B cells and IRA B cells **(F)** in pleural space 1, 2, 3 and 4 days after i.t. infection of 5x10^6^ CFU of *E. coli* or PBS (n=5-7 mice per time point). **(G)** Number of CFUs in pleural space fluid 6h and 12h after i.t. infection of 5x10^6^ CFU of *E. coli* (n=6). Data are pooled data from at least 2 independent experiments. Data represent mean ± S.E.M. and were analyzed by the Dunnett’s multiple comparisons test. *p<0.05; **p<0.01; ***p<0.001.

Next, we investigated how *E. coli* airway infection affects the pleural space. Compared to steady-state, a massive neutrophil recruitment and increased TNFα and interleukin-6 (IL-6) levels were observed in the PS from the first day of *E. coli* infection followed by a progressive decrease over time (**Figures 2A, B**) indicating a robust inflammation of the PS during the early phase of bacterial airway infection. The level of inflammation, characterized by the recruitment of neutrophils, was dependent on the intensity of the infection. Whereas only few neutrophils could be detected in the PS during steady-state, the neutrophil numbers increased proportionately to the intensity of *E. coli* infection (**Figure 2C**). We also observed a significant reduction in the numbers of large resident macrophages (LRMs) and T cells in the PS during the first days of infection and a progressive increase of inflammatory macrophages, monocytes and IRA B cells (**Figures 2D–F**). No difference was however observed in pleural B cell numbers (**Figure 2F**). Collectively, these results indicated that *E. coli* airway infection induces inflammation of the PS. Interestingly, bacteria could be detected in the pleural space from 6h post-infection (**Figure 2G**) suggesting that PS inflammation was driven by the presence of bacteria in the pleural space during *E. coli* airway infection. Mice intranasally infected with HSV-1 did not exhibit any differences in the number of neutrophils and resident macrophages in PS when compared to uninfected mice (**Figure S2A**). As well, no difference was observed in the percentage of zombie^+^ pleural LRMs (**Figure S2A**). By contrast, mice displayed increased number of neutrophils in the lungs upon HSV-1 infection (**Figure S2B**) indicating that HSV-1 did induce pulmonary inflammation. Altogether, these data suggest that inflammation of the PS seems to be restricted to bacterial infection.

### GM-CSF-Expressing B Cells Contribute Partially to GM-CSF-Induced PS Inflammation

**Figure 3:**
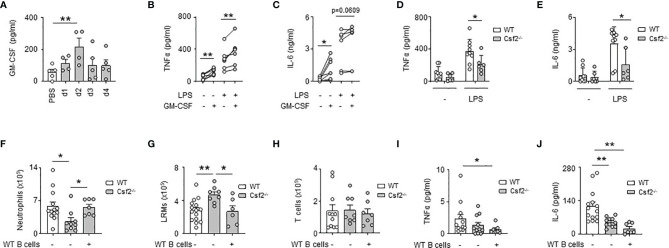
GM-CSF-expressing B cells contribute partially to GM-CSF-induced PS inflammation. **(A)** Levels of GM-CSF in pleural space 1, 2, 3 and 4 days after i.t. infection of 5x10^6^ CFU of *E. coli* or PBS (n=4-5 mice per time point). **(B, C)** Levels of TNFα **(B)** and IL-6 **(C)** in culture supernatant of pleural immune cells from naive mice 24h after *ex vivo* stimulation with GM-CSF in presence or in absence of LPS (n=5 mice). **(D, E)** Levels of TNFα **(D)** and IL-6 **(E)** in culture supernatant of pleural immune cells from naive WT or *Csf2^-/-^* mice 24h after *ex vivo* stimulation with LPS (n=6-9 mice). **(F–H)** Enumeration of neutrophils **(F)**, LRMs **(G)** and T cells **(H)** in pleural space of WT or *Csf2^-/-^* mice 24h after intrapleural injection of LPS. WT and *Csf2^-/-^* mice received PBS or WT pleural B cells into the pleural space at the same time of LPS injection (n=7-13 mice). **(I, J)** Levels of TNFα **(I)** and IL-6 **(J)** in pleural space of WT or *Csf2^-/-^* mice 24h after intrapleural injection of LPS. WT and *Csf2^-/-^* mice received PBS or WT pleural B cells into the pleural space at the same time of LPS injection (n=7-13 mice). Data are pooled data from at least 2 independent experiments. Data represent mean ± S.E.M. and were analyzed by the two-tailed paired or unpaired t-test or by the Dunnett’s multiple comparisons test. *p<0.05; **p<0.01.

We previously reported that bacterial airway infection resulted in the generation of GM-CSF^+^ IRA B cells in the pleural space (13). Considering the inflammatory activity of GM-CSF (26), we wondered whether B cell-derived GM-CSF, and in higher extent GM-CSF, might promote PS inflammation during bacterial airway infection. Upon *E. coli* airway infection, GM-CSF was secreted into the pleural space, its level increasing progressively during the first days of infection (**Figure 3A**). The *ex vivo* stimulation of pleural immune cells by recombinant GM-CSF resulted in increased TNFα and IL-6 levels in culture supernatant (**Figures 3B, C**). The same effect was also observed when pleural immune cells were concomitantly activated by LPS (**Figures 3B, C**). As well, reduced TNFα and IL-6 levels were observed in culture supernatants of pleural immune cells from *Csf2^-/-^* stimulated *ex vivo* by LPS during 24h as compared to pleural immune cells from WT mice (**Figures 3D, E**). *In vivo*, *Csf2^-/-^* mice exhibited reduced neutrophil numbers and TNFα and IL-6 levels in the PS compared to WT mice 24h after intra-pleural (i.pls.) injection of LPS (**Figures 3F, I, J**). *Csf2^-/-^* displayed also higher LRM numbers but the same amount of T cells in the PS (**Figures 3G, H**). Interestingly, the adoptive transfer of pleural WT B cells into the PS of *Csf2^-/-^* reversed the reduced number of neutrophils and increased number of LRMs observed in the pleural space of *Csf2^-/-^* 24h after i.pls. injection of LPS (**Figures 3F, G**). However, the adoptive transfer of pleural WT B cells into the PS of *Csf2^-/-^* had no effect on the pleural levels of TNFα and IL-6 (**Figures 3I, J**). Altogether, these results indicate that the ability of GM-CSF to potentiate PS inflammation is partially dependent to IRA B cells.

### *E. coli*-Induced Pleural Large Resident Macrophage Necroptosis Is Followed by Local Macrophage Proliferation

**Figure 4:**
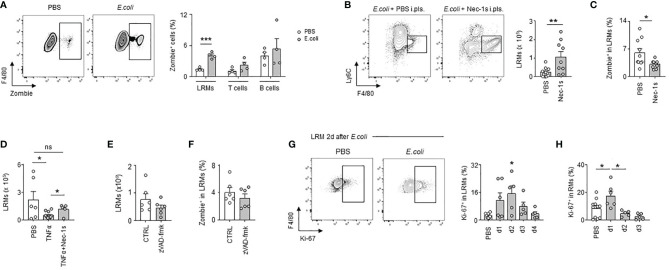
*E. coli*-induced pleural large resident macrophage necroptosis is followed by local macrophage proliferation. **(A)** Representative dot plots for LRMs and percentage of death of LRMs, T cells and B cells in their respective population 24h after i.t. infection of 5x10^6^ CFU of *E. coli* (n=4). **(B)** Representative dot plots and enumeration of LRM in pleural space 24h after i.t. infection of 5x10^6^ CFU of *E. coli* and i.pls. injection of 100µg of necrostatin-1s (Nec-1s) or PBS (PBS) (n=9 mice). **(C)** Percentage of death of LRMs in the LRM population 24h after i.t. infection of 5x10^6^ CFU of *E. coli* and i.pls. injection of 100µg of necrostatin-1s (Nec-1s) or PBS (PBS) (n=11 mice). **(D)** Enumeration of LRM in pleural space 24h after i.pls. injection of 100ng of recombinant TNFα with and without i.pls. injection of 100µg of necrostatin-1s (n=6-12 mice). **(E)** Enumeration of LRMs in pleural space 24h after i.t. infection of 5x10^6^ CFU of *E. coli* and i.pls. injection of 100µg of zVAD-fmk or control (CTRL) (n=6 mice). **(F)** Percentage of death of LRMs in the LRM population 24h after i.t. infection of 5x10^6^ CFU of *E. coli* and i.pls. injection of 100µg of zVAD-fmk or control (CTRL) (n=6 mice). **(G)** Representative dot plots and percentage of pleural Ki-67^+^ LRM in the LRM population during steady-state and 1, 2, 3 and 4 after i.t. infection of 5x10^6^ CFU of *E. coli* (n=5-7 mice per time point). **(H)** Percentage of Ki-67^+^ LRM in the LRM population during steady-state and 1, 2 and 3 days after i.n. injection of LPS (n=5-11). Data are pooled data from at least 2 independent experiments. Data represent mean ± S.E.M. and were analyzed by the two-tailed unpaired t-test or by the Dunnett’s multiple comparisons test. *p<0.05; **p<0.01; ***p<0.001.

During the first days of infection, we observed a significant reduction in the numbers of LRMs, the more abundant RMs in pleural cavity (**Figure 2E**). A similar decrease of pleural LRMs was detected after i.pls. injection of LPS (**Figure S3A**). No significant difference was observed in the numbers of macrophages in lungs and BALF 24h after i.pls. injection of LPS (**Figure S3B**) suggesting an absence of RM emigration to these compartments. While we observed increased pleural LRM death in PS 24h after *E. coli* infection, no significant difference was observed in the death of pleural T cells and B cells indicating that cell death observed in PS during *E. coli* infection was specific to macrophages (**Figure 4A**). Flow cytometry analysis did not reveal any differences in the percentage of early apoptotic LRMs (Annexin-V^+^ 7AAD^-^) in the PS 24h after i.t. infection with *E. coli* when compared to controls (**Figure S4**). Only the percentage of late apoptotic/necroptotic LRMs (Annexin-V^+^ 7AAD^+^) in PS increased in mice infected with *E. coli* (**Figure S4**). Interestingly, the pleural injection of necrostatin-1s (Nec-1s), a chemical inhibitor of necroptosis (27), resulted in increased numbers of LRMs 24h after *E. coli* infection (**Figure 4B**) as well as a reduced LRM death (**Figure 4C**) when compared to controls. Likewise, the number of LRMs was reduced 24h after the pleural injection of TNFα, a cytokine described to promote necroptosis (28), this effect being reversed with the concomitant intra-pleural injection of Nec-1s (**Figure 4D**). However, no significant difference was observed in the number of LRMs and the percentage of LRM death after pleural injection of zVAD-fmk (**Figures 4E, F**), a chemical pan-caspase inhibitor, indicating the death of pleural LRMs observed during *E. coli* infection was only associated to necroptosis. The reduction of pleural LRMs was followed by a progressive replenishment over time (**Figure 2E**). The analysis of the proliferation marker Ki-67 by flow cytometry revealed increased percentages of Ki-67^+^ LRMs at day 1, 2 and 3 post-infection and 24h after intra-nasal injection of LPS, suggesting a local proliferation of LRMs in the PS (**Figures 4G, H**). Altogether, these results suggest that *E. coli* airway infection induces early necroptosis of pleural LRMs, which is sustained by their local proliferation.

### Necroptotic Pleural Resident Macrophages Promote Pleural Space Inflammation Upon Bacterial Pneumonia

**Figure 5:**
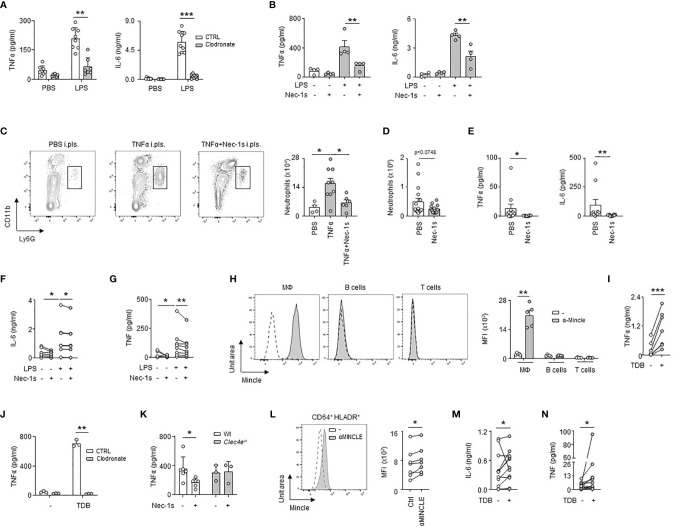
Necroptotic pleural resident macrophages promote pleural space inflammation upon bacterial pneumonia. **(A)** Levels of TNFα and IL-6 in culture supernatant of pleural cells depleted (clodronate) or not (CTRL) of RM 24h after *ex vivo* stimulation with LPS. Mice received i.pls. injection of liposome control (CTRL) or clodronate (Clodronate) 2 days before harvesting pleural cells (n=7-9). **(B)** Levels of TNFα and IL-6 in culture supernatant of pleural cells from WT mice 24h after *ex vivo* stimulation with LPS (2µg/ml) and/or Nec-1s (50µM) (n=4 mice). **(C)** Representative dot plots and enumeration of neutrophils in pleural space 24h after i.pls. injection of 100ng of recombinant TNFα with and without i.pls. injection of 100µg of necrostatin-1s (n=4-9). **(D)** Enumeration of neutrophils in pleural space 24h after i.t. infection of 5x10^6^ CFU of *E. coli* and i.pls. injection of 100µg of necrostatin-1s (Nec-1s) or PBS (PBS) (n=10 mice). **(E)** TNFα and IL-6 levels in pleural space 24h after i.t. infection of 5x10^6^ CFU of *E. coli* and i.pls. injection of 100µg of necrostatin-1s (Nec-1s) or PBS (PBS) (n=8-10 mice). **(F, G)** Levels of IL-6 **(F)** and TNF **(G)** in the supernatants of human PEF cells stimulated for 24h with LPS and/or Nec-1s (n=8 out of 24). **(H)** Representative histogram and mean fluorescence intensity (MFI) of Mincle at the surface of pleural CD11b^+^F4/80^+^ macrophages, B cells and T cells in naive mice (n=5 mice). **(I)** TNFα levels in culture supernatant of pleural cells from naive mice 24h after *ex vivo* stimulation with TDB (n=7). **(J)** Levels of TNFα in culture supernatant of pleural cells depleted (clodronate) or not (CTRL) in RM 24h after *ex vivo* stimulation with TDB. Mice received i.pls. injection of liposome control (CTRL) or clodronate (Clodronate) 2 days before harvesting pleural cells (n=3). **(K)** TNFα levels in culture supernatant of pleural cells from naive WT or *Clec4e^-/-^* mice 24h after *ex vivo* stimulation with LPS and Nec-1s (n=3-6). **(L)** Representative histogram and MFI of MINCLE at the surface of human CD64^+^HLA-DR^+^ PEF cells (n=7 of 24). **(M, N)** Levels of IL-6 **(M)** and TNF **(N)** in supernatants of human PEF cells stimulated with or without TDB for 24h (n=11 out of 24). Data are pooled data from at least 2 independent experiments. Data represent mean ± S.E.M. and were analyzed by the two-tailed paired or unpaired t-test or by the Tukey’s multiple comparisons test. *p<0.05; **p<0.01; ***p<0.001.

We then investigated the impact of LRM necroptosis during airway infection. We observed that the induction of TNFα and IL-6 observed 24h after the *ex vivo* stimulation of pleural cells by LPS was significantly reduced in the presence of Nec-1s, as well as after the *ex vivo* stimulation of RM-depleted pleural cells by clodronate liposomes (**Figures 5A, B**). Likewise, the pleural injection of Nec-1s resulted in the reduction of neutrophil influx into the PS 24h after *E. coli* infection or after i.pls. injection of recombinant TNFα (**Figures 5C, D**). The elevated levels of TNFα and IL-6 observed in the PS one day after *E. coli* airway infection or after *ex vivo* stimulation of human pleural effusion fluid (PEF) cells by LPS were also reduced in the presence of Nec-1s (**Figures 5E–G** and **Table S1**). All together, these results indicate that necroptosis of pleural RMs promotes PS inflammation upon bacterial airway infection. Necroptotic cells induce inflammation by releasing Damage-Associated Molecular Pattern (DAMPs) in the microenvironment (29), which can be recognized by receptors such as the C-type lectin Mincle (20). Mincle was found to be expressed in pleural immune cells, its expression increasing 1 day after *E. coli* airway infection (**Figure S5A**). The naive pleural immune cell compartment mainly consists of RM, serosal B1 B cells and T cells, however, flow cytometric analysis revealed that only RM expressed Mincle (**Figure 5H**), with a higher expression after *E. coli* airway infection (**Figure S5B**). Neutrophils recruited into the PS during *E. coli* infection expressed also Mincle on their surface (**Figure S5C**). The *ex vivo* stimulation of naive pleural immune cells with a Mincle agonist (Trehalose-6,6-dibehenate, TDB) induced the phosphorylation of Syk, a protein involved in Mincle signaling, in LRMs as well as the secretion of TNFα in the culture supernatant (**Figures 5I** and **S5D**). Likewise, the pleural injection of TDB resulted in increased influx of neutrophils into the PS and high levels of pleural TNFα when compared to control mice (**Figures S5E, F**). Interestingly, the induction of TNFα observed 24h after *ex vivo* stimulation with TDB was abolished when pleural cells were depleted in resident macrophages by clodronate liposomes or after the stimulation of pleural cells from *Clec4e*-deficient mice (**Figures 5J** and **S5G**). Thus, these results indicate that the activation of Mincle on pleural resident macrophages promote inflammation. We next investigated if necroptotic LRMs might promote inflammation through Mincle. We found that whereas the secretion of TNFα observed 24h after LPS *ex vivo* stimulation of pleural cells from naive WT mice was reduced in presence of Nec-1s, no difference was observed in *Clec4e^-/-^* mice (**Figure 5K**) indicating a role of Mincle in necroptosis-induced inflammation. In humans, MINCLE was found expressed at the surface of pleural CD64^+^ HLADR^+^ macrophages (**Figure 5L**) and PEF cells exhibited higher IL-6 and TNF levels than controls after *ex vivo* stimulation by TDB (**Figures 5M, N**). Altogether, these results suggest that Mincle expressed by pleural RMs contributes to PS inflammation by recognizing DAMPs released by necroptotic RM during *E. coli* airway infection.

### PS Inflammation Results in Enhanced Lung Immunity and in a Better Protection

**Figure 6:**
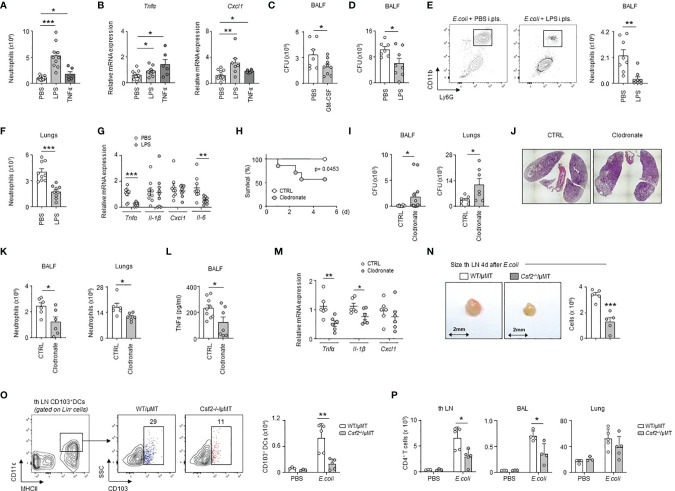
PS cells enhance lung immunity upon bacterial airway infection. **(A, B)** Enumeration of neutrophils **(A)** and relative mRNA expression of *Tnfα* and *Cxcl1* **(B)** in lungs 24h after i.pls. injection of PBS, 20µg of LPS or 100ng of TNFα (n=6-14 mice). **(C)** Number of CFU in BALF 6h after i.t. *E. coli* infection with (grey) or without (white) concomitant i.pls. injection of GM-CSF (n=7-9). **(D–G)** Mice were sacrificed 6h after i.t. *E. coli* infection with (grey) or without (white) concomitant i.pls. injection of LPS. **(D)** Number of CFU in BALF (n=9). **(E, F)** Enumeration of neutrophils in BALF **(E)** and lungs **(F)** (n=9). **(G)** Relative mRNA expression of *Tnf*α, *Il-1β*, *Cxcl1* and *Il-6* in lungs (n=8-9 mice). (**H–M**) Mice received i.pls. injection of liposome control (white) or clodronate (grey) and 2 days later they were intra-tracheally infected by *E. coli* (5x10^6^ CFU) during 5 days **(H)** or 24h **(I–M)**. **(H)** Survival curve (n=7-8 mice/group). Data were analyzed by Log-rank (Mantel-Cox) test. **(I)** Number of CFU in BALF (n=12 mice) and lungs (n=7 mice) 24h post-infection. **(J)** Representative hematoxylin and eosin staining in lung sections 24h post-infection (n=4 mice). **(K)** Enumeration of neutrophils in BALF and lungs 24h post-infection (n=6 mice). **(L)** TNFα level in BALF 24h post-infection (n=7-9 mice). **(M)** Relative mRNA level expression of *Tnf*α, *Il-1β*, *Cxcl1* in lungs 24h post-infection (n=6 mice). (**N**) Enumeration of cells in tracheobronchial lymph node of chimeric WT/µMT and Csf2^-/-^/µMT mice 4 days after i.t. *E. coli* infection (n=5). **(O)** Gating strategy and enumeration of CD103^+^ DCs in tracheobronchial lymph node of chimeric WT/µMT and Csf2^-/-^/µMT mice 4 days after i.t. *E. coli* infection (n=3-5). **(P)** Enumeration of CD4^+^ T cells in tracheobronchial lymph node, BAL and lung of chimeric WT/µMT and Csf2^-/-^/µMT mice 4 days after i.t. *E. coli* infection (n=3-5). **(B, G, M)** The expression level was arbitrarily set to 1 for one sample from the PBS/CTRL group, and the values for the other samples were calculated relatively to this reference. Data are pooled data from at least 2 independents experiments. Data represent mean ± S.E.M. and were analyzed by the two-tailed unpaired t-test. *p<0.05; **p<0.01; ***p<0.001.

To test whether inflammation of the PS might modulate the pulmonary immune response, we induced inflammation in the pleural space by intra-pleural injection of LPS or recombinant TNFα. We observed increased numbers of neutrophils and an increased mRNA expression of the pro-inflammatory genes *Tnfα*, *Il-1β* and *Cxcl1* in lungs 1 day after the induction of inflammation as compared to controls (**Figures 6A, B** and **S6A**). As well, mice displayed an increased number of neutrophils in lungs after pleural injection of TDB (**Figure S6B**). Those data suggest therefore that early PS inflammation might induce protection during airway infection. Considering that the lungs include a component of the PS (the visceral pleura), we cannot exclude that the mRNA data might reflect in part the level of activation of the visceral pleura. However, mice that received pleural administration of GM-CSF or LPS at the onset of *E. coli* airway infection displayed reduced bacterial burden in the BALF 6h post-infection when compared to controls (**Figures 6C, D**) confirming then the protective effect of PS inflammation. The reduced bacterial burden observed in mice treated with LPS was associated to a reduced pulmonary inflammation as shown by a lower neutrophil number in BALF and lungs and a decreased mRNA expression of the pro-inflammatory genes *Tnfα* and *Il-6*, but not *Il-1β* and *Cxcl1*, in lungs (**Figures 6E**–**G**). By contrast, the specific depletion of pleural macrophages by intra-pleural injection of clodronate liposomes (**Figures S7A**–**D**) resulted in reduced survival and increased bacterial burden in the BALF and lungs one day after *E. coli* airway infection when compared to controls (**Figures 6H, I**). Although lung histology did not reveal major differences (**Figure 6J**), mice that received i.pls. injection of clodronate exhibited reduced number of neutrophils in BALF and lungs (**Figure 6K**), a decrease of BALF TNFα levels (**Figure 6L**) and a decrease of the mRNA expression of the pro-inflammatory cytokines *Tnfα*, *Il-1β* and *Cxcl1* in the lungs (**Figure 6M**) suggesting that pleural macrophages strengthen the pulmonary immune response during *E.coli* infection. Having observed that IRA B cells develop in the pleural space upon airway infection (**Figure 2F**); that B cell-derived GM-CSF promotes PS inflammation (**Figures 3F**–**J**); and that B cell-derived GM-CSF promotes innate immunity by orchestrating IgM secretion (13), we wondered if pleural IRA B cells might also modulate the adaptive immune response. Mice with a B cell restricted deficiency to produce GM-CSF (*Csf2^-/-^*/µMT) showed reduced cell numbers in tracheobronchial lymph node 4 days post-infection compared to WT/µMT control mice (**Figure 6N**). Interestingly, *Csf2^-/-^*/µMT mice had reduced numbers of CD103^+^ dendritic cells in tracheobronchial lymph node 4 days post-infection suggesting a reduced T cell activation (**Figure 6O**). Indeed, *Csf2^-/-^*/µMT mice had a reduced number of CD4^+^ T cells in tracheobronchial lymph node as well as in lungs and BAL 4 days post-infection (**Figure 6P**). Collectively, our results suggest that pleural space protects against *E. coli* airway infection by potentiating both innate and adaptive immunity.

## Discussion

Airway infection remains the most common cause of infection-related mortality, with *E. coli* representing 11,8% of bacterial pneumonia from patients hospitalized in Europe and the Mediterranean region (30). During pneumonia, the pleural inflammation may result in pleural effusion (PE). As the mortality increases in patients who develop severe PE (10), pleural inflammation is thought to be detrimental. For this reason, most studies aimed to decipher the mechanisms involved in the generation of PE in order to assess anti-inflammatory effects of new pharmaceutical agents (11, 12). Considering that pleural inflammation is seen as the consequence of a strong pulmonary inflammation, its role during immune responses against airway infection is still poorly understood. Here, we show that, beyond its detrimental effect, PS inflammation can be, in fact, beneficial during airway infection by boosting pulmonary immunity.

GM-CSF is a hematopoietic growth factor produced by both hematopoietic and nonhematopoietic cells that plays an important role in many inflammatory diseases as shown by the different clinical trials targeting GM-CSF or its receptor (26). During Gram^-^ lung infection, mice deficient in GM-CSF showed an increased mortality and bacterial burden in lungs (31), this protective effect relying in part on the ability of B cell-derived GM-CSF to orchestrate IgM immunity (13) and on GM-CSF to improve alveolar macrophage function (31). In this study we describe new mechanisms associated to GM-CSF-promoted protection against bacterial pneumonia. We observed that pleural GM-CSF contributed to PS inflammation resulting in improved innate and adaptive immune responses. Our results let suggest that GM-CSF potentiates PS inflammation by amplifying the secretion of the inflammatory cytokine TNFα and IL-6, by promoting the accumulation of neutrophils in PS and by potentiating LRM necroptosis. We cannot exclude that GM-CSF might also synergize with TNFα as previously described in rheumatoid arthritis (32).

We also found that necroptosis of pleural LRM contributed to PS inflammation. Necroptosis can be triggered after ligand binding to death receptors (e.g. TNF-receptor-1, Fas) or pathogen recognition receptors (e.g. TLR3, TLR4) (28). Our study reveals that necroptosis of pleural LRM can be promoted by LPS and TNFα. It has also been shown that type I interferon-induced necroptosis in macrophages during *S. Thyphimurium* infection mediates the phosphorylation of RIP-1 (33). Considering that pleural resident macrophage necroptosis was inhibited by Nec-1s, an inhibitor of RIP-1, we cannot exclude a possible contribution of type I interferons in pleural macrophage necroptosis. This is strengthened by the presence of B cells in the pleural space, a cell type described to produce type I IFN after bacterial infection (34). The release of Damage-Associated Molecular Pattern (DAMPs) by necroptotic cells induces inflammation in the microenvironment (29), which are sensed by many receptors such as Mincle (35). Mincle is an inducible receptor expressed on myeloid cells (36) that plays an important role during bacterial pneumonia (21, 37). At steady-state, we observed that Mincle was only expressed by pleural RM and its expression increased upon *E.coli* infection. Mincle induction can be mediated by LPS, a compound associated to Gram negative bacteria such as *E.coli* and by the pro-inflammatory cytokines TNFα and IL-6, cytokines overexpressed in PS during *E.coli* infection (19). The activation of Mincle by microbial or endogenous ligands from damaged and necrotic cells results in the production of TNFα (38), a cytokine characterized to be a strong inducer of necroptosis (28). Our data suggest therefore the existence of an amplifying loop for LRM necroptosis during *E. coli* infection: the release of DAMPs by necroptotic LRM stimulates Mincle^+^ cells to secrete TNFα, which intensifies LRM necroptosis, a phenomenon previously reported *in vitro* using bone marrow-derived macrophages (39). Moreover, the description of Dectin-1 (40) and TLR2/4 expression (41) in the PS suggest that Mincle is probably not the only DAMPs-sensing receptor involved during *E. coli* airway infection.

The ability of lung parenchyma and PS to stimulate each other suggests the existence of an immune-physiological communication loop, in which the PS possibly helps the lung parenchyma to mount an effective immune response against bacterial infection. Our previous work showed that pleural immune cells do migrate to the lungs during pneumonia (13). Thus, the movement of activated pleural immune cells to the lungs might potentiate the airway immune response through the secretion of pro-inflammatory molecules leading either to increased leukocyte recruitment or to improved leukocyte function. However, we also cannot exclude that the increase of the pulmonary immune response might result from heightened systemic inflammatory response due to the inflammatory molecules secreted by the PS cells. Pleural inflammation is thought to be detrimental as the mortality increases in patients who develop severe pleural effusion (10). Therefore, the role of the PS during pulmonary infection seems to depend on the intensity of its inflammation. An excessive inflammation has a detrimental effect characterized by impaired breathing through limited lung expansion as observed in patients with a large para-pneumonic effusion or empyema (42), whereas an early and optimal inflammation has a beneficial effect by enhancing immunity.

The ability of necroptosis to improve immune responses has already been described in a model of skin infection, sepsis, liver infection or after vaccinia virus infection (43–45). However, necroptosis has also been shown to have detrimental effects in lungs during infection with the virulent *S.aureus* strain MRSA USA300 (46) or with a high dosage of LPS (47). The macrophage subset involved in necroptosis therefore seems to define the type of response: pleural resident macrophages and Kupffer cell (44) necroptosis has beneficial effects during infection whereas alveolar macrophage necroptosis (46) has a detrimental effect. Collectively, our study reveals an unexpected function of pleural space inflammation on enhancing pulmonary immunity upon bacterial airway infection.

## Data Availability Statement

The original contributions presented in the study are included in the article/**Supplementary Material**, further inquiries can be directed to the corresponding author.

## Ethics Statement

The studies involving human participants were reviewed and approved by local ethics review board of the University of Erlangen (UKER 10_16 B; UKER 339_15 Bc; UKER 56_12B; DRKS-ID: DRKS00005376). The patients/participants provided their written informed consent to participate in this study. The animal study was reviewed and approved by the animal review committee from the university hospital Dresden and Erlangen and the local governmental animal committee.

## Author Contributions

AB conceived the project, designed the experiments, performed experiments, analyzed and interpreted data, and wrote the manuscript. MP, IK, BK, and EN performed experiments. AJ, FC, DT, and HS provided human samples. CK, MB, RL, and RG helped to interpret the data and edited the manuscript. GW conceived and supervised the project, designed the experiments, performed experiments, analyzed and interpreted data, and wrote the manuscript. All authors contributed to the article and approved the submitted version.

## Funding

This work was supported by the German research foundation WE4892/3-1, WE4892/4-1, WE4892/8-1 and WE4892/9-1 (to GW), and BE6981/1-1 (to AB).

## Conflict of Interest

The authors declare that the research was conducted in the absence of any commercial or financial relationships that could be construed as a potential conflict of interest.

## Publisher’s Note

All claims expressed in this article are solely those of the authors and do not necessarily represent those of their affiliated organizations, or those of the publisher, the editors and the reviewers. Any product that may be evaluated in this article, or claim that may be made by its manufacturer, is not guaranteed or endorsed by the publisher.

## Supplementary Material

Supplementary Figure 1 Specific depletion of pleural space cells after Talc treatment. **(A–D)** Enumeration of leukocytes in pleural space **(A)**, lungs **(B)**, broncho-alveolar lavage fluid **(C)**, and blood **(D)** of mice 7 days after i.pls. injection of talc using the ICAPS technique (n=4). **(E)** Relative mRNA expression of *Tnfα* in lungs 7 days after i.pls. injection of talc using the ICAPS technique (n=4). The expression level was arbitrarily set to 1 for one sample from the PBS group, and the values for the other samples were calculated relatively to this reference. Data are pooled data from 2 independent experiments. Data represent mean ± S.E.M. and were analyzed by the two-tailed unpaired t-test. *p<0.05; **p<0.01; ***p<0.001.

Supplementary Figure 2 HSV-1 pneumonia does not induce PS inflammation. **(A)** Enumeration of neutrophils and LRMs and percentage of Zombie^+^ LRMs in the LRM population in the PS 24h after i.n. injection of PBS or 7.5x10^6^ PFU of HSV-1 (n=4 mice). **(B)** Enumeration of neutrophils in lungs 24h after i.n. injection of PBS or 7.5x10^6^ PFU of HSV-1 (n=4 mice). Data are pooled data from at least 2 independent experiments. Data represent mean ± S.E.M. and were analyzed by the two-tailed unpaired t-test. ***p<0.001.

Supplementary Figure 3 The reduction of resident macrophages is specific to the PS. **(A)** Enumeration of large resident macrophages (LRM) in pleural space 1, 2 and 3 days after i.pls. injection of LPS (20µg) or PBS (n=4-11 mice per time point). **(B)** Enumeration of macrophages in lungs (left) and BALF (right) 24h after i.pls injection of 20 µg of LPS or PBS (n=6 mice). Data are pooled data from at least 2 independent experiments. Data represent mean ± S.E.M. and were analyzed by the two-tailed unpaired t-test. *p<0.05; **p<0.01; ***p<0.001.

Supplementary Figure 4
*E. coli* pneumonia does not induce early apoptotic LRMs. Representative dot plots and percentage of early apoptotic cells (Annexin-V^+^ 7AAD^-^) and late apoptotic/necroptotic cells (Annexin-V^+^ 7AAD^+^) inside the LRM population in the PS of mice infected or not with *E. coli* during 24h (n=4). Data are pooled data from at least 2 independent experiments. Data represent mean ± S.E.M. and were analyzed by the two-tailed unpaired t-test. *p<0.05.

Supplementary Figure 5 Stimulation of pleural cells by TDB induces inflammation. **(A)** Relative mRNA expression of *Clec4e* in PS cells 24h after i.t. injection of PBS or 5x10^6^ CFU of *E. coli* (n=4 mice). **(B)** MFI of Mincle at the surface of pleural CD11b^+^F4/80^+^ macrophages 24h after i.t. injection of PBS or 5x10^6^ CFU of *E. coli* (n= 7 mice). **(C)** Representative histogram and MFI of Mincle at the surface of pleural neutrophils 24h after i.t. injection of 5x10^6^ CFU of *E. coli* (n= 7 mice). **(D)** Representative histogram and MFI of phospho-Syk in pleural CD11b^+^F4/80^+^ macrophages 30min after *ex vivo* stimulation with TDB (10µg) (n=3). **(E, F)** Enumeration of neutrophils **(E)** and TNFα levels **(F)** in PS 24h after i.pls. injection of PBS or TDB (50µg) (n=9 mice). **(G)** Levels of TNFα in culture supernatants of pleural immune cells from WT or *Clec4e^-/-^* mice 24h after *ex vivo* stimulation with TDB (10µg/ml) (n=2-3). **(A)** The expression level was arbitrarily set to 1 for one sample from the PBS group, and the values for the other samples were calculated relatively to this reference. Data are pooled data from at least 2 independent experiments. Data represent mean ± S.E.M. and were analyzed by the two-tailed paired or unpaired t-test. *p<0.05; **p<0.01; ***p<0.001.

Supplementary Figure 6 Specific pleural space stimulation induces pulmonary immune response. **(A)** Relative mRNA expression of *Il-1β* in lungs 24h after i.pls. injection of PBS, 20µg of LPS or 100ng of TNFα (n=6-14 mice). The expression level was arbitrarily set to 1 for one sample from the PBS group, and the values for the other samples were calculated relatively to this reference. **(B)** Enumeration of neutrophils in lungs 24h after i.pls. injection of PBS or TDB (n=7-9 mice). Data are pooled data from at least 2 independent experiments. Data represent mean ± S.E.M. and were analyzed by the two-tailed unpaired t-test. *p<0.05; **p<0.01; ***p<0.001.

Supplementary Figure 7 Specific depletion of pleural space macrophages after clodronate treatment. **(A–D)** Mice received i.pls. injection of liposome control (white) or clodronate (grey) and 2 days later they were sacrificed. **(A)** Enumeration of CD115^+^ F4/80^+^ macrophages and neutrophils in PS. **(B)** Percentage of CD115+ F4/80+ macrophages in PS. **(C, D)** Enumeration of CD115+ F4/80+ macrophages in lungs **(C)** and BALF **(D)**. Data are pooled data from at least 2 independent experiments. Data represent mean ± S.E.M. and were analyzed by the two-tailed unpaired t-test. *p < 0.05; **p < 0.01; ***p < 0.001.
