# Supplementary figures and images for: Pleural Resident Macrophages and Pleural IRA B Cells Promote Efficient Immunity Against Pneumonia by Inducing Early Pleural Space Inflammation
Source: Front Immunol. 2022 Apr 14;13:821480. doi: 10.3389/fimmu.2022.821480 (PMC9047739; doi:10.3389/fimmu.2022.821480)

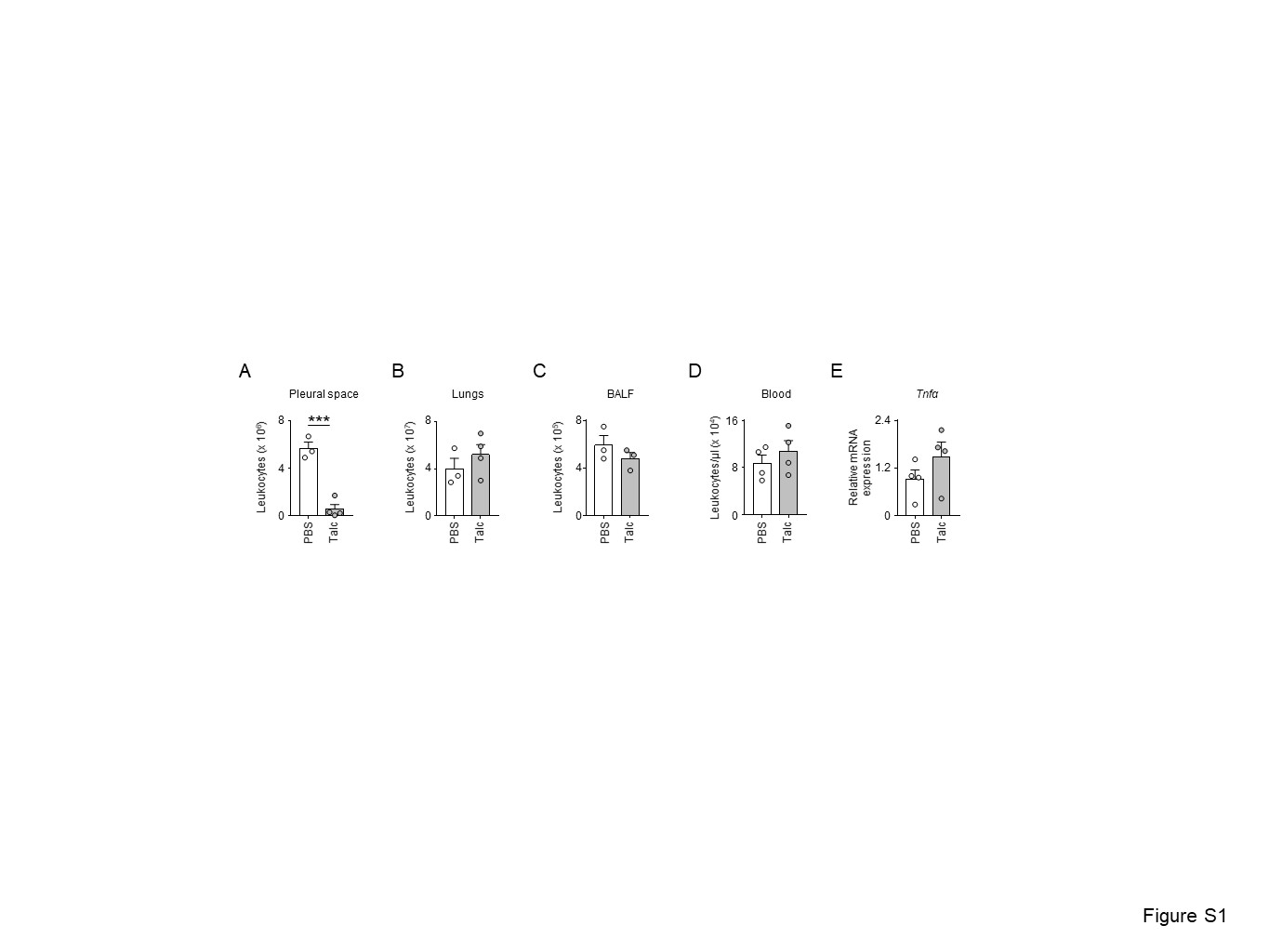

Supplement: Supplementary Figure 1 — Specific depletion of pleural space cells after Talc treatment. (A–D) Enumeration of leukocytes in pleural space (A), lungs (B), broncho-alveolar lavage fluid (C), and blood (D) of mice 7 days after i.pls. injection of talc using the ICAPS technique (n=4). (E) Relative mRNA expression of Tnfα in lungs 7 days after i.pls. injection of talc using the ICAPS technique (n=4). The expression level was arbitrarily set to 1 for one sample from the PBS group, and the values for the other samples were calculated relatively to this reference. Data are pooled data from 2 independent experiments. Data represent mean ± S.E.M. and were analyzed by the two-tailed unpaired t-test. *p<0.05; **p<0.01; ***p<0.001. [file Image_1.jpeg]

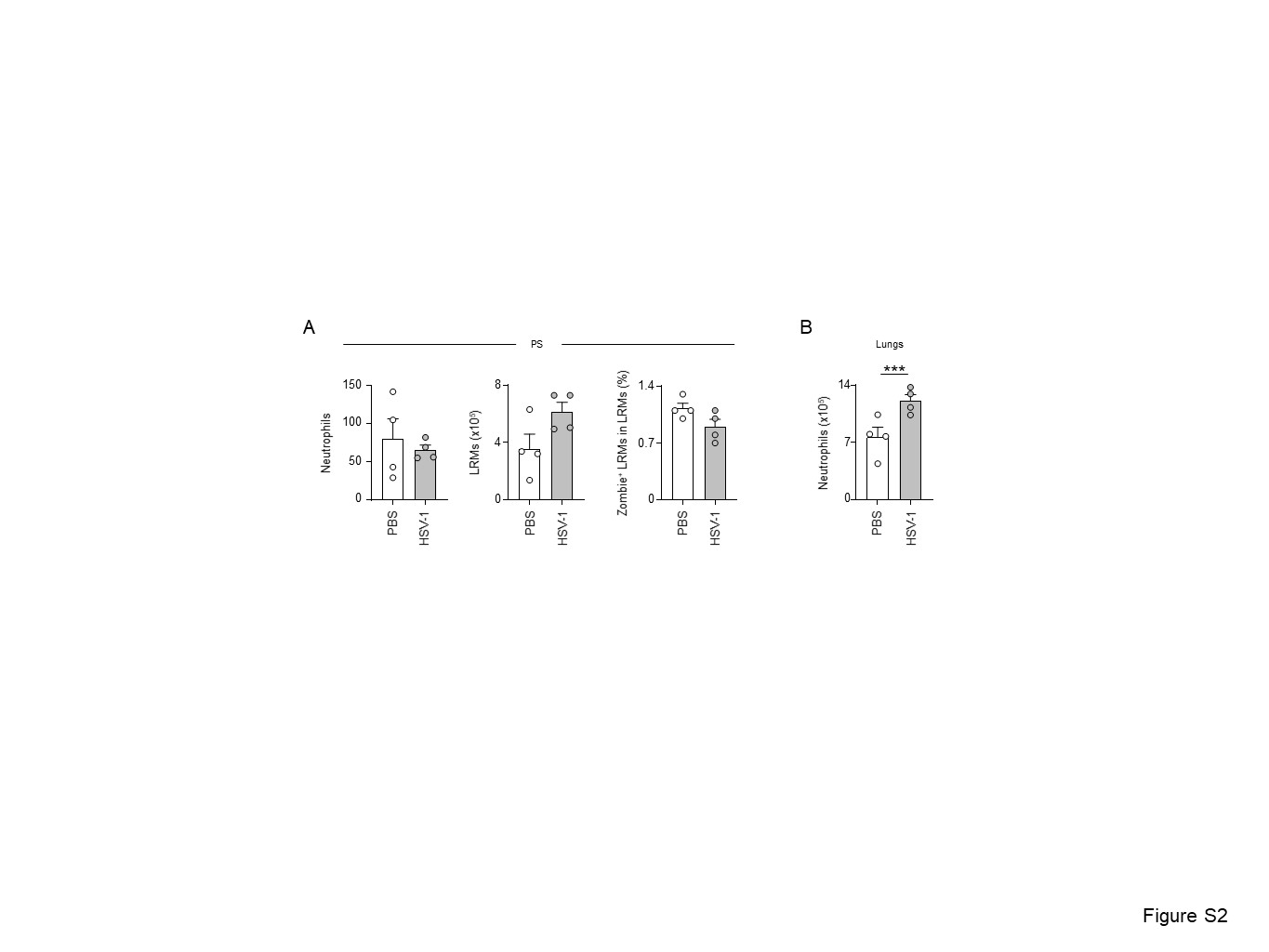

Supplement: Supplementary Figure 2 — HSV-1 pneumonia does not induce PS inflammation. (A) Enumeration of neutrophils and LRMs and percentage of Zombie+ LRMs in the LRM population in the PS 24h after i.n. injection of PBS or 7.5x10^6^ PFU of HSV-1 (n=4 mice). (B) Enumeration of neutrophils in lungs 24h after i.n. injection of PBS or 7.5x10^6^ PFU of HSV-1 (n=4 mice). Data are pooled data from at least 2 independent experiments. Data represent mean ± S.E.M. and were analyzed by the two-tailed unpaired t-test. ***p<0.001. [file Image_2.jpeg]

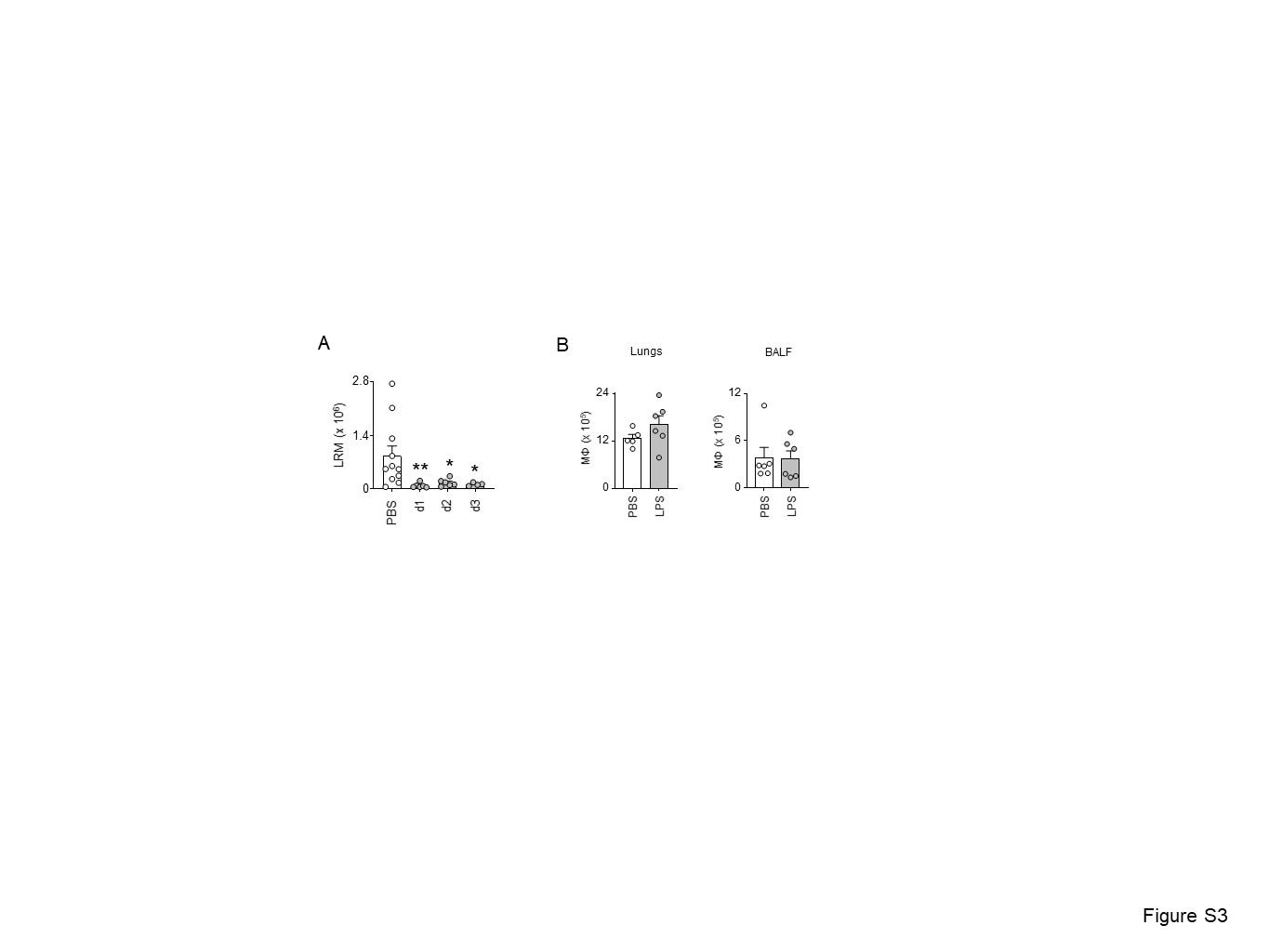

Supplement: Supplementary Figure 3 — The reduction of resident macrophages is specific to the PS. (A) Enumeration of large resident macrophages (LRM) in pleural space 1, 2 and 3 days after i.pls. injection of LPS (20µg) or PBS (n=4-11 mice per time point). (B) Enumeration of macrophages in lungs (left) and BALF (right) 24h after i.pls injection of 20 µg of LPS or PBS (n=6 mice). Data are pooled data from at least 2 independent experiments. Data represent mean ± S.E.M. and were analyzed by the two-tailed unpaired t-test. *p<0.05; **p<0.01; ***p<0.001. [file Image_3.jpeg]

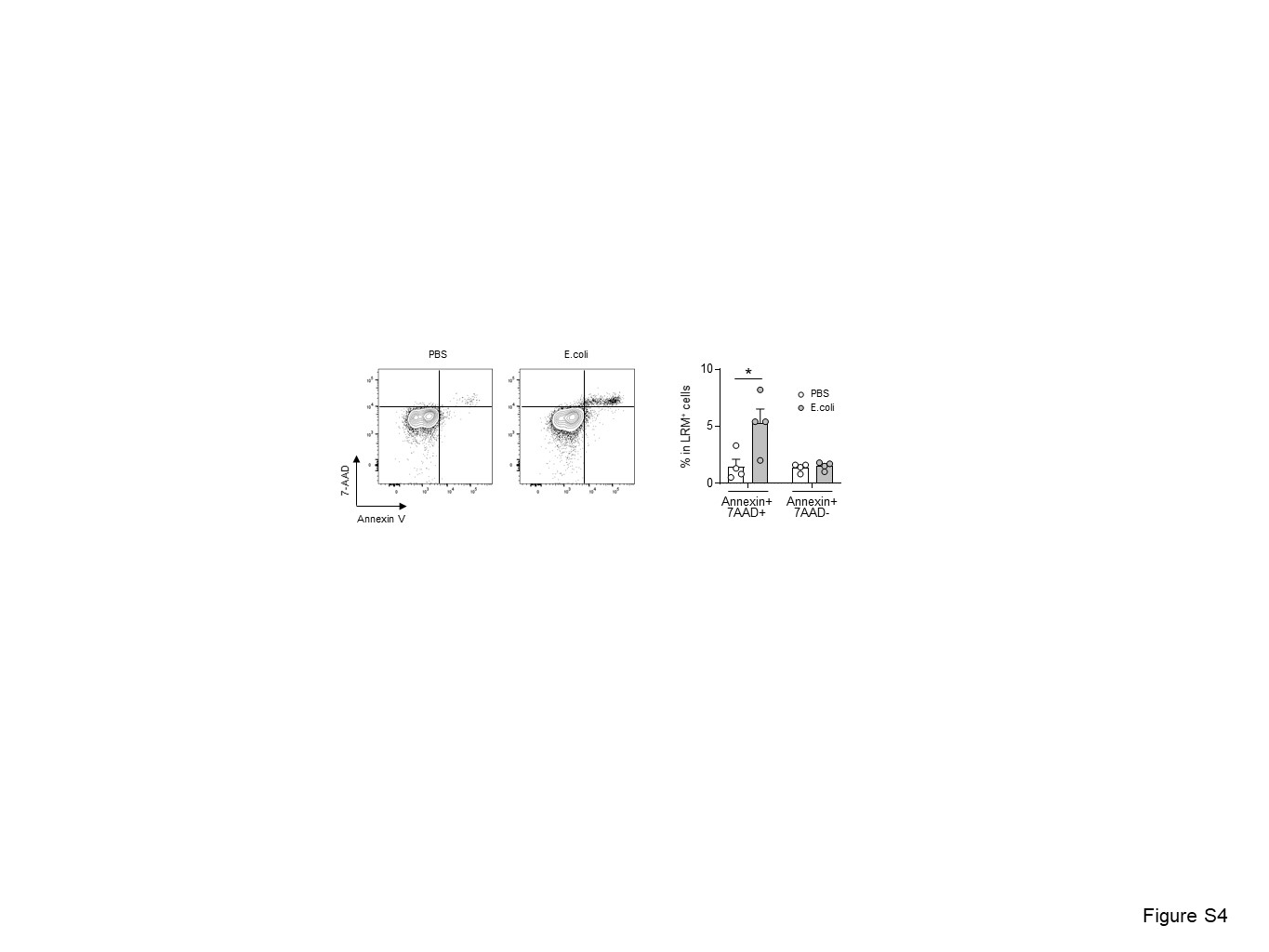

Supplement: Supplementary Figure 4 — E. coli pneumonia does not induce early apoptotic LRMs. Representative dot plots and percentage of early apoptotic cells (Annexin-V+ 7AAD-) and late apoptotic/necroptotic cells (Annexin-V+ 7AAD+) inside the LRM population in the PS of mice infected or not with E. coli during 24h (n=4). Data are pooled data from at least 2 independent experiments. Data represent mean ± S.E.M. and were analyzed by the two-tailed unpaired t-test. *p<0.05. [file Image_4.jpeg]

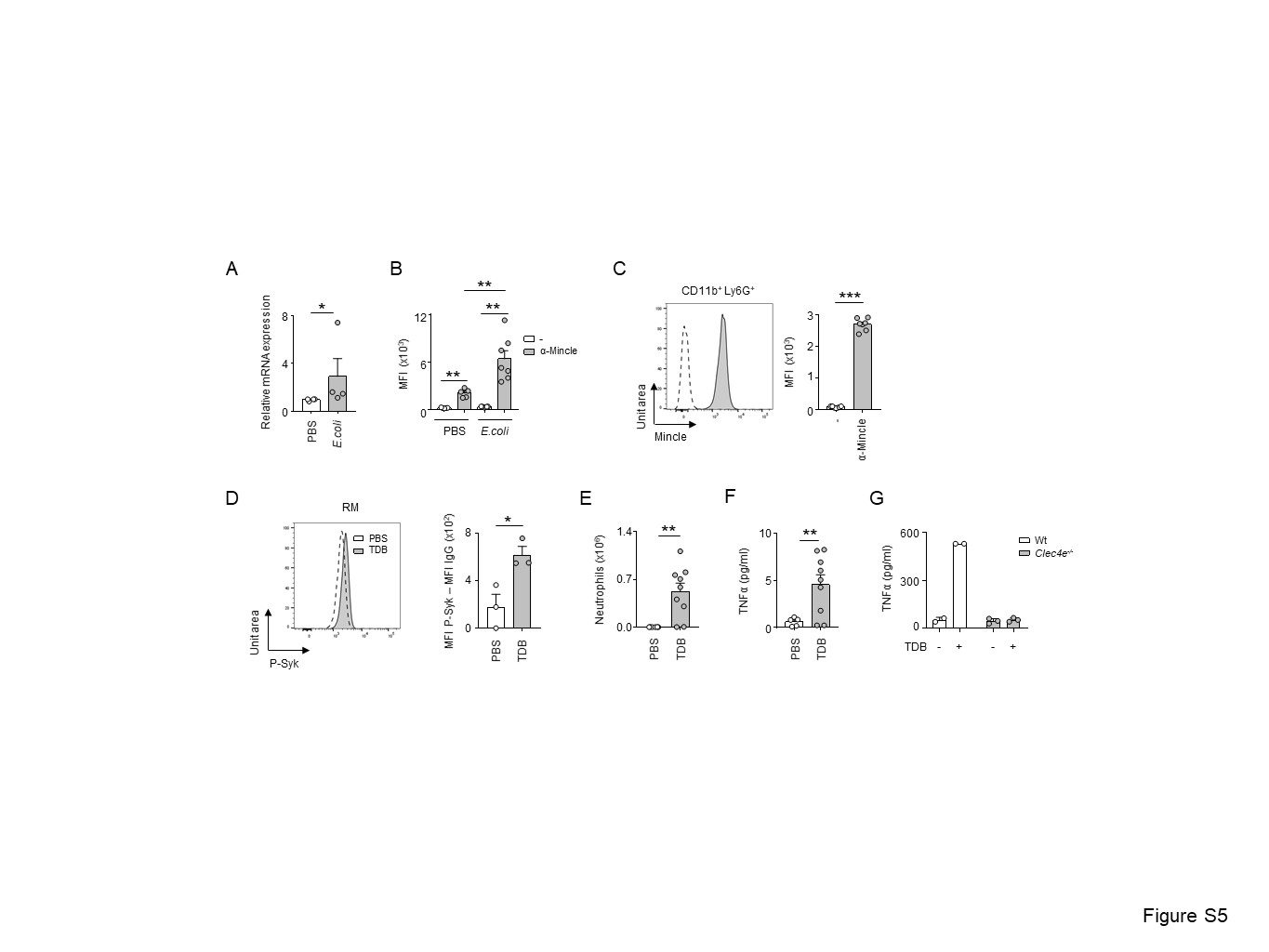

Supplement: Supplementary Figure 5 — Stimulation of pleural cells by TDB induces inflammation. (A) Relative mRNA expression of Clec4e in PS cells 24h after i.t. injection of PBS or 5x10^6^ CFU of E. coli (n=4 mice). (B) MFI of Mincle at the surface of pleural CD11b+F4/80+ macrophages 24h after i.t. injection of PBS or 5x10^6^ CFU of E. coli (n= 7 mice). (C) Representative histogram and MFI of Mincle at the surface of pleural neutrophils 24h after i.t. injection of 5x10^6^ CFU of E. coli (n= 7 mice). (D) Representative histogram and MFI of phospho-Syk in pleural CD11b+F4/80+ macrophages 30min after ex vivo stimulation with TDB (10µg) (n=3). (E, F) Enumeration of neutrophils (E) and TNFα levels (F) in PS 24h after i.pls. injection of PBS or TDB (50µg) (n=9 mice). (G) Levels of TNFα in culture supernatants of pleural immune cells from WT or Clec4e-/- mice 24h after ex vivo stimulation with TDB (10µg/ml) (n=2-3). (A) The expression level was arbitrarily set to 1 for one sample from the PBS group, and the values for the other samples were calculated relatively to this reference. Data are pooled data from at least 2 independent experiments. Data represent mean ± S.E.M. and were analyzed by the two-tailed paired or unpaired t-test. *p<0.05; **p<0.01; ***p<0.001. [file Image_5.jpeg]

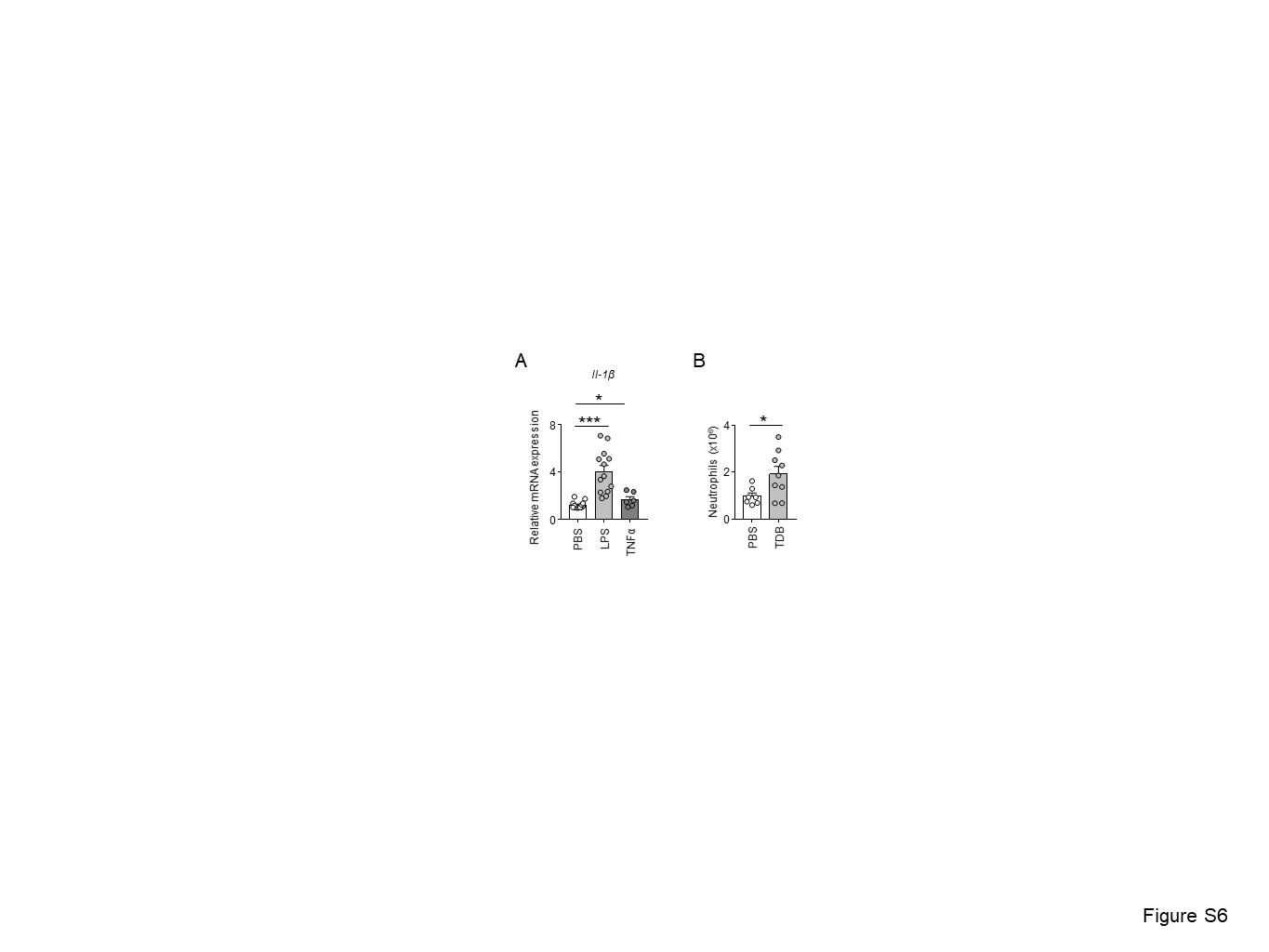

Supplement: Supplementary Figure 6 — Specific pleural space stimulation induces pulmonary immune response. (A) Relative mRNA expression of Il-1β in lungs 24h after i.pls. injection of PBS, 20µg of LPS or 100ng of TNFα (n=6-14 mice). The expression level was arbitrarily set to 1 for one sample from the PBS group, and the values for the other samples were calculated relatively to this reference. (B) Enumeration of neutrophils in lungs 24h after i.pls. injection of PBS or TDB (n=7-9 mice). Data are pooled data from at least 2 independent experiments. Data represent mean ± S.E.M. and were analyzed by the two-tailed unpaired t-test. *p<0.05; **p<0.01; ***p<0.001. [file Image_6.jpeg]

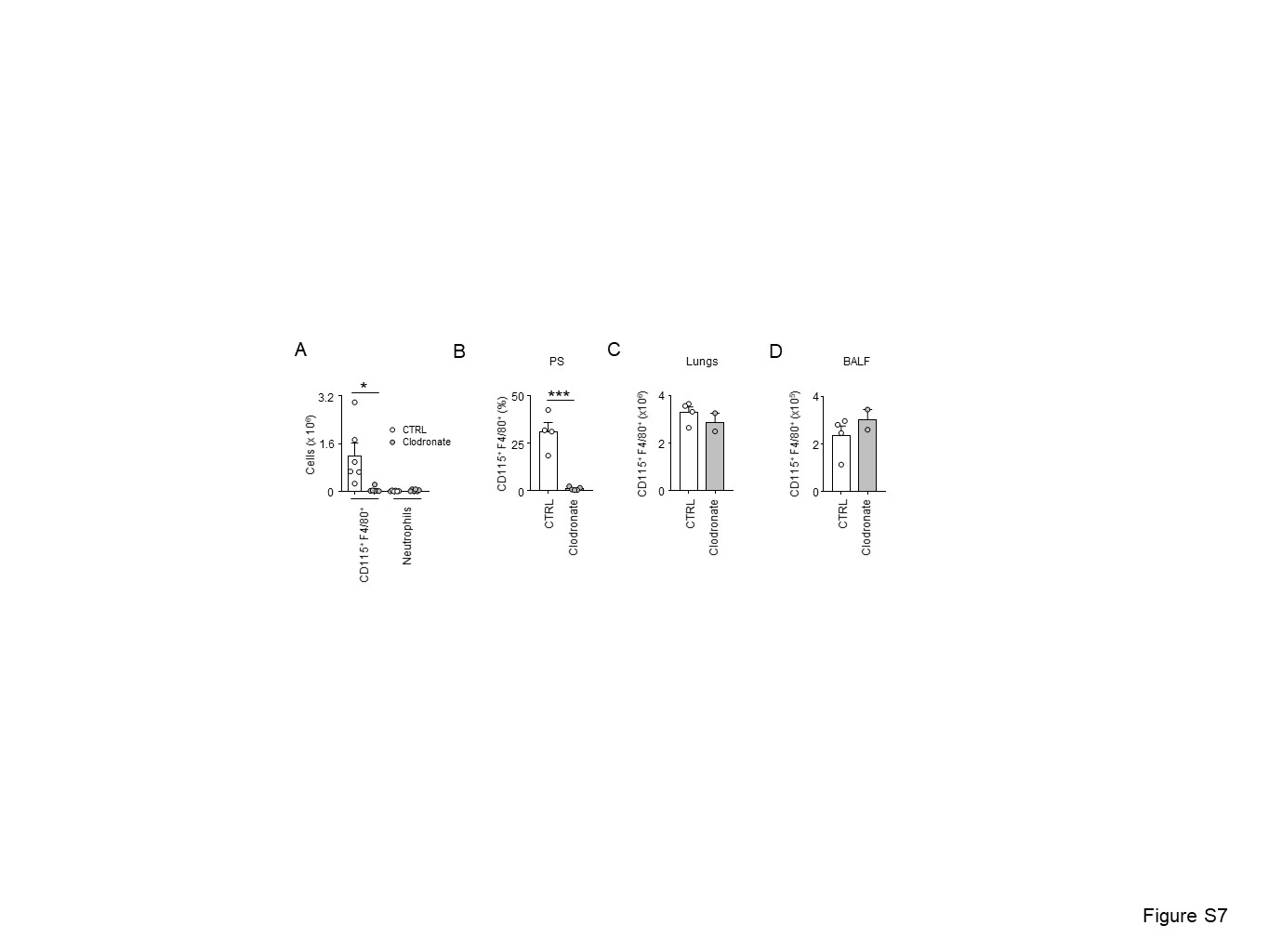

Supplement: Supplementary Figure 7 — Specific depletion of pleural space macrophages after clodronate treatment. (A–D) Mice received i.pls. injection of liposome control (white) or clodronate (grey) and 2 days later they were sacrificed. (A) Enumeration of CD115+ F4/80+ macrophages and neutrophils in PS. (B) Percentage of CD115+ F4/80+ macrophages in PS. (C, D) Enumeration of CD115+ F4/80+ macrophages in lungs (C) and BALF (D). Data are pooled data from at least 2 independent experiments. Data represent mean ± S.E.M. and were analyzed by the two-tailed unpaired t-test. *p < 0.05; **p < 0.01; ***p < 0.001. [file Image_7.jpeg]
